# Point-of-Care Ultrasound in the Pediatric Intensive Care Unit

**DOI:** 10.3389/fped.2021.830160

**Published:** 2022-02-01

**Authors:** Luke Burton, Vidit Bhargava, Michele Kong

**Affiliations:** Division of Critical Care, Department of Pediatrics, School of Medicine, University of Alabama, Birmingham, AL, United States

**Keywords:** POCUS, point of care, ultrasound, pediatrics, critical care

## Abstract

Ultrasonography has been widely used in medicine for decades but often by specific users such as cardiologists, obstetricians, and radiologists. In the last several years, the use of this imaging modality has moved to the bedside, with clinicians performing and interpreting focused point of care ultrasonography to aid in immediate assessment and management of their patients. The growth of point of care ultrasonography has been facilitated by advancement in ultrasound-related technology and emerging studies and protocols demonstrating its utility in clinical practice. However, considerable challenges remain before this modality can be adopted across the spectrum of disciplines, primarily as it relates to training, competency, and standardization of usage. This review outlines the history, current state, challenges and the future direction of point of care ultrasonography specifically in the field of pediatric critical care medicine.

## Introduction

Point-of-care ultrasound (POCUS) describes the acquisition and interpretation of images by the treating clinician, the end-user, at the bedside (1). It allows for real-time, data-informed clinical decisions, without dependence on a specialist to obtain the images or to interpret them. In pediatric critical care, this ultrasound framework lends itself perfectly as it allows for procedures to be done safely and for rapid, convenient serial reassessments aimed at improving diagnosis and monitoring (2).

In the past decade, significant advancements have been made in pediatric critical care POCUS (3). However, as with most significant advancements, it is not without controversy. The rise of POCUS has been rapid, and many questions remain unanswered, including those related to competency and training. Usage can alter workflow, increase the financial burden, and incorrect interpretations made by inadequately trained users can pose significant risks to patients. However, when POCUS is used as a supplement to existing clinical aids, or as an extension of the physical exam, rather than an independent tool to overrule or replace other diagnostic modality, its benefits are immense, and can provide critical information and guidance in taking care of our patient (4, 5).

In this review, we discuss the origins, current state and evolution of POCUS within pediatric critical care, as well as the future direction and the obstacles that must be overcome to continue its advancement.

## History of Critical Care Point-of-Care Ultrasound

Medical ultrasound was derived from World War I SONAR technology and then adapted by radiology, cardiology, and obstetrics over the ensuing decades. The first case series of 150 critically ill patients, demonstrating the utility of POCUS was published by Lichtenstein and Axler in 1993 (6), POCUS altered the therapeutic plan in up to one quarter of these patients. However, early point-of-care machines provided poor image quality and were cumbersome to move around and operate, limiting the widespread use of POCUS. For the next two decades, ultrasound machines became smaller, less expensive, more portable and allowed for improved image quality (7). Table 1 gives an overview of the different ultrasound probes that are currently being used, and their general applications. As the technology advanced, POCUS developed rapidly in parallel, largely spearheaded by adult emergency medicine and critical care (8).

**Table 1:** Ultrasound probes and their general applications.

**Probe**	**Configuration**	**Frequency (MHz)**	**Applications**
Standard linear	Long, narrow rectangular probe face	5–13	To visualize superficial structures (such as the pleural space, vascular structures and soft tissue) and for procedural guidance)
Neonatal/pediatric linear	Long, narrow rectangular probe face or hockey stick configuration	7–22	Same as standard linear with a smaller footprint for procedural purposes and for a better fit in between rib spaces
Phased array	Small, square probe face	1–5	For visualization of cardiac anatomy and abdominal compartment
Neonatal/pediatric phased array	Smaller square/rectangular probe face	4–8	Same as standard phased array but with smaller footprint for a better fit in between rib spaces
Curvilinear	Curved/rectangular probe face	1–5	Allows for a deeper penetration with a wide field of view. Used for visualization of the abdominal and thoracic space, as well as for procedural guidance

The earliest application of POCUS in pediatric critical care was for central venous catheter placement (9). The transition to ultrasound guided vascular access was motivated by the recommendations from the Agency for Healthcare Research and Quality (AHRQ) as one of the twelve most highly rated practices to prevent medical errors (10). The safety and efficacy of ultrasound guided central venous cannulation allowed for expansion of procedural guidance to arterial line placement, lumbar puncture, peritoneal, pleural, and even pericardial fluid drainage (11). Today, ultrasound guidance in performing procedures is widely accepted and practiced.

On the other hand, diagnostic POCUS implementation and adoption into practice remains variable. A national survey of 128 academic pediatric critical care units in the United States confirmed low and variable rates of implementation, mostly from a lack of user training, competence and confidence (12). In 2014, pediatric POCUS pioneers issued a call to action for responsible and widespread implementation into practice (13). The first reported pediatric critical care focused institutional POCUS training program was implemented in 2015 (14). Since then, POCUS education, training and clinical application have improved in both pediatric critical care and emergency medicine (15). Finally, the growth of pediatric POCUS is also evident in the development of practice guidelines. Although guidelines were published for adult patients by the Society of Critical Care Medicine in 2015, (16, 17) pediatric recommendations have been limited until recently, when the European Society for Pediatric and Neonatal Intensive Care (ESPNIC) published comprehensive, evidence-based guidelines for pediatric intensivists (3).

## Current State of Critical Care Point-of-Care Ultrasound

The following sections discuss an overview of the various diagnostic and procedural POCUS applications specific to the pediatric critical care and their impact on patient management.

### Procedural Ultrasound

Pediatric critical care practice relies heavily on diagnostic and therapeutic procedures. Utilization of bedside imaging in performing procedures improves accuracy, overall success, and patient satisfaction. It decreases time to successful completion of the procedure and complications (18, 19).

#### Vascular Cannulation

**Figure 1:**
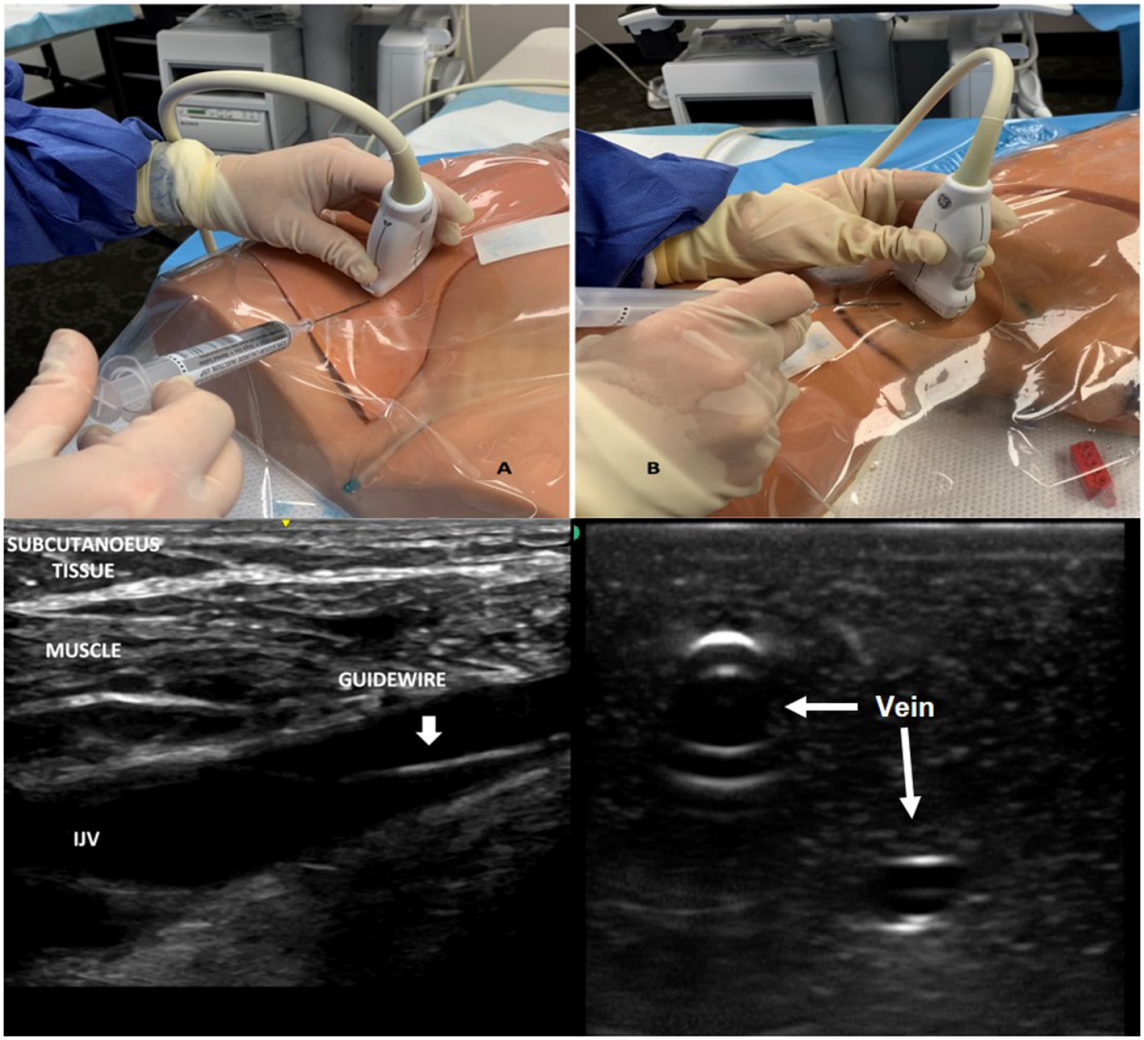
Image demonstrating vascular imaging in short and longitudinal access and probe placement for obtaining images in the two planes. **(A)** Shows probe placement and imaging in longitudinal axis. **(B)** Demonstrating probe placement and imaging in short axis.

Central line placement is a commonly performed procedure in the pediatric intensive care unit (PICU). Ultrasound guidance for internal jugular central line placement is the current standard of care in pediatric and adult patients (20). Ultrasound guidance for central venous cannulation increases overall success rate, decreases number of mean attempts required and arterial punctures, especially for internal jugular vein cannulation (18, 21). The evidence is limited for femoral vein and subclavian vein cannulation but supports similar improvement in success and decrement in complications when ultrasound guidance is used (21). The two most common orthogonal planes of central venous cannulation are longitudinal (in plane) and transverse (out-of-plane) (Figure 1). There is no consensus as to the superiority of either approach. Vezzani et al. in their study of adult cardiac patients undergoing subclavian cannulation by an experienced anesthesiologist, reported superiority of transverse approach. The cannulation in transverse approach group was associated with higher overall success, first puncture success and lower time to cannulation, failed attempts, and complications (22). In contrast, trained emergency medicine physicians in a simulation study found the longitudinal approach to be superior (23). Currently, there are no pediatric studies comparing the superiority of the two approaches.

Ultrasound guidance has also been shown to aid in peripheral venous access. Vinograd et al. demonstrated that use of ultrasound guided peripheral intravenous access in children was associated with increased first attempt success rates and increased line longevity compared with traditional placement technique (24). Similarly, Peripherally Inserted Central Catheter (PICC) placed with ultrasound guidance resulted in increased first attempt success rate, overall success rate, and decreased procedure time (25). POCUS can also be used to confirm catheter tip location in venous vascular cannulation. Ultrasound confirmed catheter tip location has been deemed to be safe and effective in midline catheters in adults, umbilical venous catheter and PICC lines in neonates, and to have good agreement with chest x-ray in central venous catheters in pediatric patients (26–29).

For arterial cannulation, there is limited but high-quality evidence (5 RCT's) in pediatric patients suggesting that ultrasound guidance improves first attempt success rate (risk ratio 1.96; 95% CI 1.34–2.85) and decreases complications (risk ratio 0.20; 95% CI 0.07–0.60) (19).

Although concerns were raised regarding the decrease in proficiency of landmark-based methods after adoption of ultrasound by trainees, they were not substantiated by a recent prospective observational study (30). Lastly, there is also skepticism regarding the utility of ultrasound guidance in the hands of experienced providers. In a study by Froehlich et al., ultrasound guided cannulation by experienced physicians were not superior to landmark guided placement even though a robust improvement was noticed in the resident and fellow group (31).

#### Pleural and Peritoneal Drainage

Thoracentesis, paracentesis, chest tubes, and abdominal drains are important diagnostic and therapeutic procedures performed frequently in the PICU using both real-time and static ultrasound guidance. These procedures are discussed below in greater detail in the abdominal and lung ultrasound sections.

#### Lumbar Puncture

Lumbar puncture is another commonly performed procedure in the PICU. The incidence of failed procedure and traumatic tap can be as high as 50% (32). Evidence suggesting the utility of ultrasound guidance in lumbar puncture especially for infants and neonates is rapidly emerging. It is a feasible approach to identifying landmarks and interspaces prior to performing the lumbar puncture (33). Ultrasound guidance increased the overall number of successful taps, while decreasing the number of traumatic taps, shortened the time to procedure completion, caused fewer needle passes and enhanced patient satisfaction (34, 35). There was a high rate of procedural success even in patients where a previous lumbar puncture using landmarks approach had been previously attempted (36).

### Cardiovascular Ultrasound

Hemodynamic instability with and without myocardial dysfunction are common in critically ill children. Focused cardiac ultrasound (FCU) helps in the rapid assessment of myocardial function, fluid status and signs of an obstructive physiology in patient with hemodynamic instability. It can be integrated with clinical assessment to differentiate the etiology of shock as well as to make decisions regarding fluid administration, vasopressor or inotrope usage, and other treatment modalities (37, 38). Although much of the early advancement in FCU was achieved in adults, cardiac images obtained in children have better resolution, encouraging the use of this tool in pediatric patients with hemodynamic instability (39). As the knowledge of expanded applications for critical care POCUS has increased, the number of critical care clinicians employing FCU has also increased (15).

#### Evaluation of Cardiac Function

**Figure 2:**
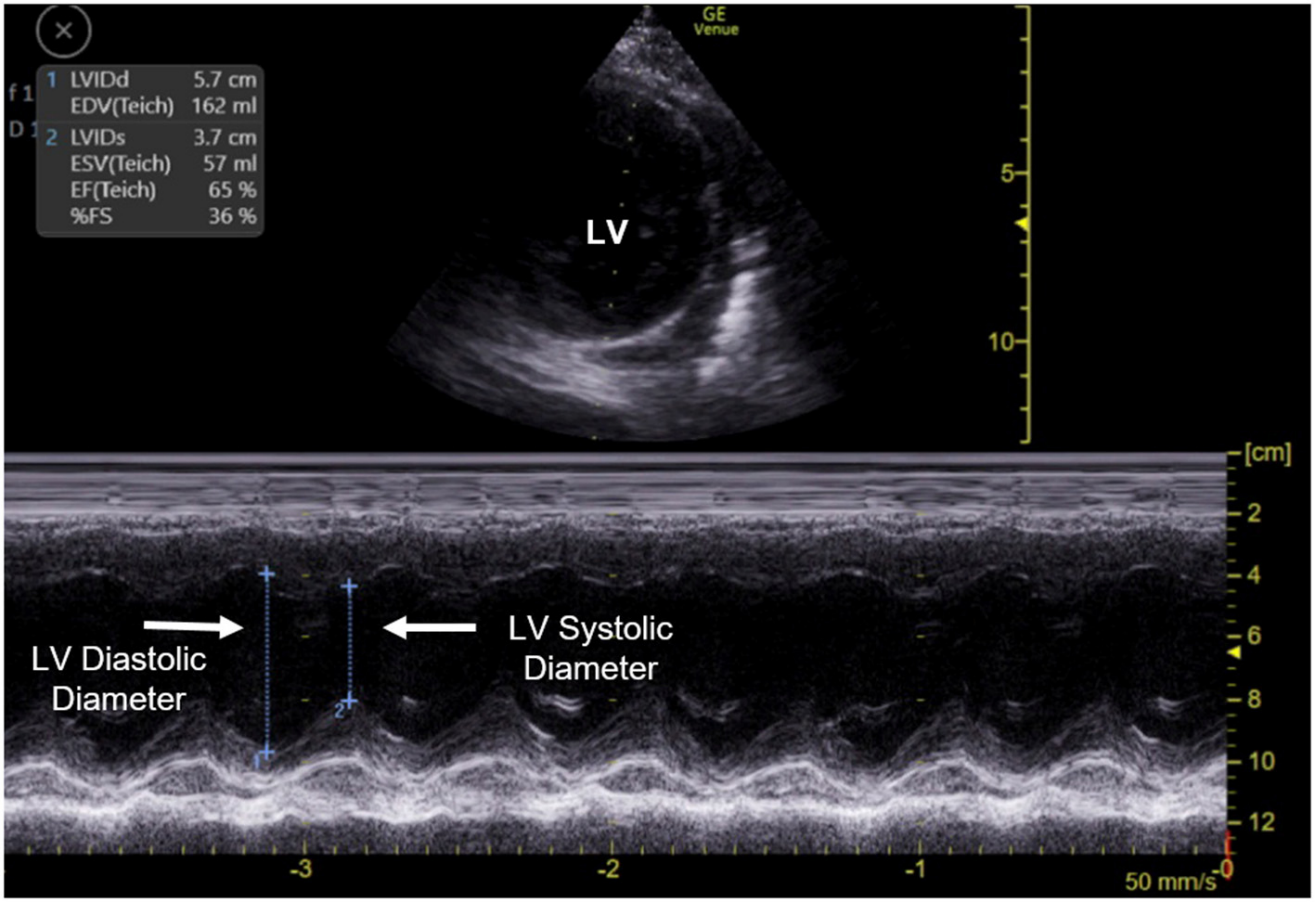
Quantitative estimation of left ventricle (LV) function using fractional shortening. The upper half of the image displays the placement of M-mode line through the left ventricle in a parasternal short axis view of the heart. The lower half of the image displays the M-mode output. Left ventricle systolic and diastolic diameters are measured to calculate fractional shortening. The scale represents the depth of imaging. LV, Left ventricle.

Critical care clinicians can examine the systolic and diastolic function of both the ventricles using FCU. While both ventricles can be examined, left ventricular systolic function is more readily assessed. In FCU, the heart is imaged in several different views and the left ventricle systolic function is “eyeballed,” or assessed qualitatively (37). Questions are often raised regarding the ability of non-cardiology clinicians to accurately assess myocardial function using echocardiography. In a study by Spurney et al., focused echocardiography performed and interpreted by non-cardiologists using a portable machine in a pediatric population yielded >90% accuracy in the assessment of ventricular function, ventricular size, and the presence or absence of pericardial effusion. Qualitative assessment of left ventricular contractility by intensivists had 96% concordance with cardiologist's interpretations for “clinically significant diagnosis” (40). Conlon et al., reported that a group of POCUS-credentialed pediatric intensivists achieved >90% agreement on ventricular function with cardiologists (38). Critical care clinicians can also employ several quantitative methods of assessing left ventricle systolic function to augment their assessment such as fractional shortening (FS), end point septal separation (EPSS) and fractional area change (FAC) (37) (Figure 2). Right ventricle function can also be assessed using FCU. However, the lack of uniform geometry and the contraction of the right ventricle in more than one axis makes it much more challenging to assess its function. Nonetheless, using several different views, the right ventricular wall motion, volume and wall thickness can be assessed, and pulmonary artery systolic pressure can be estimated (41). Guidelines recommend that critical care physicians should use qualitative and semi-quantitative measures to assess for pulmonary hypertension and right ventricle dysfunction (3). Taken together, all these studies suggest that pediatric critical care physicians can perform FCU safely.

#### Evaluation of Volume Status and Fluid Responsiveness

The accurate assessment of volume status (preload) and fluid responsiveness is challenging in critically ill pediatric patients. Although POCUS has been applied to this question in many different ways, the best application is yet to be determined. Table 2 provides an overview of volume responsiveness assessment using ultrasound guidance with suggested adult and pediatric cutoff values. The inferior vena cava (IVC) assessment using both *static* measures, in which a single view of IVC at a discrete point in time is obtained and *dynamic* measures, whereby changes in IVC size over a period of time are obtained, have been an ultrasound target for assessment of volume status for a long time. However, both the static measures and dynamic IVC measurements such as IVC collapsibility index (IVCCI) and distensibility index (IVCDI) are affected by cardiopulmonary interactions, such as in the setting of increased spontaneous breathing efforts. In critically ill patients on the mechanical ventilator, the reliability of these values is further decreased, particularly in the setting of high mean airway pressures (42, 43).

**Table 2:** Assessments of Volume Responsiveness (VR).

**Parameter**	**Definition/explanation**	**Adult cut-off suggesting VR**
IVC collapsibility index	Volume Status Assessment in spontaneously breathing patients (Min IVC diameter-Max IVC diameter)/ Max IVC diameter	>50–55%
IVC distensibility index	Volume Status Assessment in Mechanically Ventilated Patients (Max IVC diameter-Min IVC diameter)/ Min IVC diameter	>18%
Aortic flow velocity variability	Measurement of peak velocity of flow *via* pulsed wave Doppler proximal to aortic valve	>12–15% variability
Left ventricular outflow tract velocity time integral	Doppler ultrasound measurement of blood flow proximal to the aortic valve. It is measured as the area under the velocity time curve obtained from doppler waveform	≥15% variability

In adults, an IVCCI >50% predicts hypovolemia and fluid responsiveness in adults, (37) but this has not proven to be predictive of volume status or fluid responsiveness in children (37, 44). An IVCDI >18% predicts fluid responsiveness in adults with a sensitivity of 80% and a specificity of 94% (37, 45). In contrast, a recent study in mechanically ventilated children did not find IVCDI to be a predictor of fluid responsiveness, instead, the authors found a positive correlation between IVC distensibility and percent fluid overload by weight (46).

**Figure 3:**
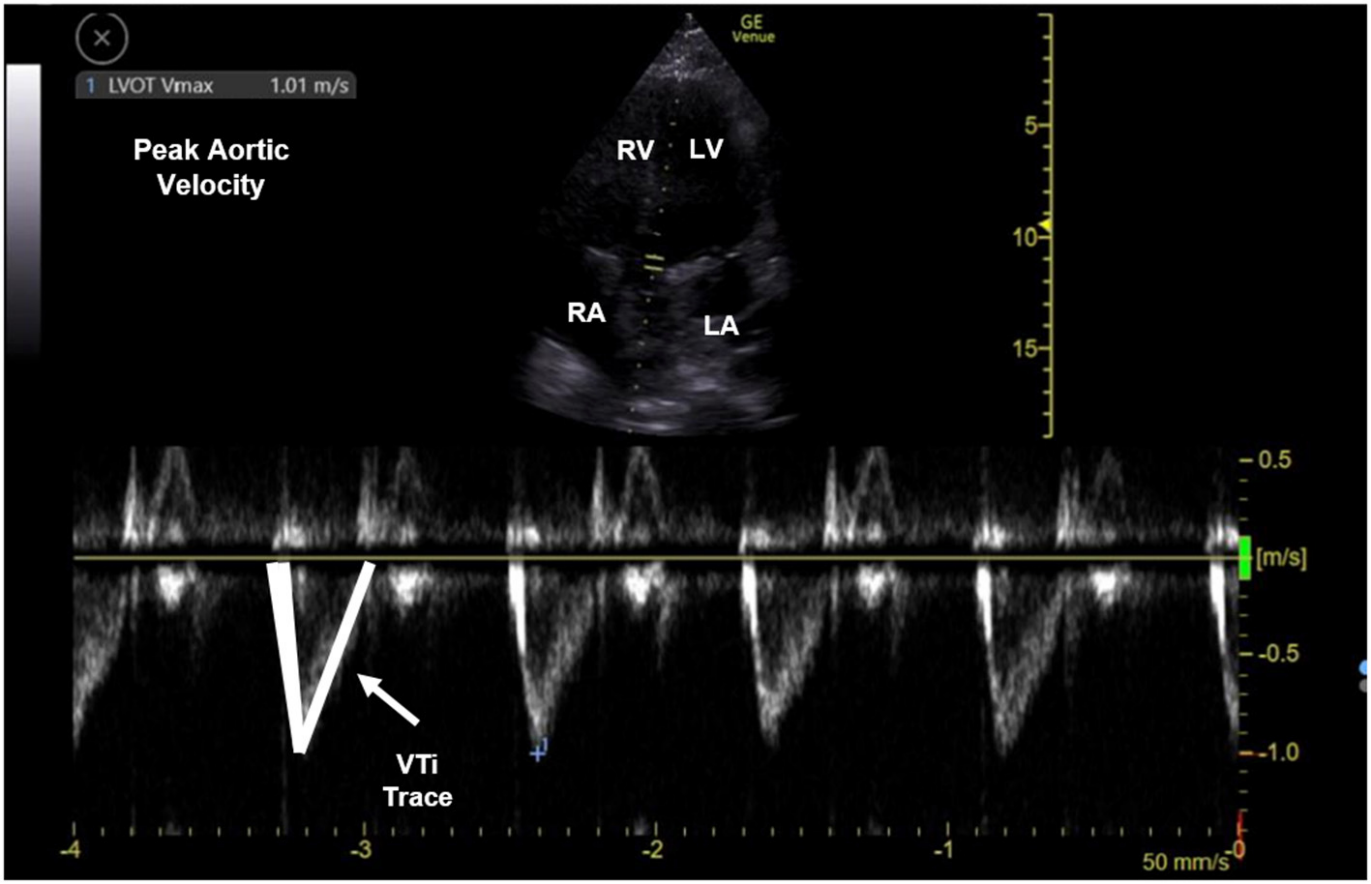
Pulse doppler waveform measuring peak aortic velocity and velocity time integral (VTi). The upper half of the image displays an apical 5 chamber view with pulse doppler gate at the aortic outflow tract. The lower half of the image displays doppler waveform (m/sec). The waveform is traced to estimate VTi and measure peak aortic velocity. RV, right ventricle; LV, left ventricle; RA, right atrium; LA, left atrium; VTi, velocity time integral.

Two other dynamic measures of fluid responsiveness, velocity time integral (VTI) and aortic flow variability (AFV) index are much more reliable measures of volume status and fluid responsiveness in children (Figure 3). They have consistently predicted fluid responsiveness in mechanically ventilated children with good sensitivity (92%) and specificity (85.5%) (47). However, these measures are technically more challenging to obtain and require a deeper understanding of the principles of doppler, limiting its clinical application.

#### Evaluation of the Pericardial Space

Pericardial Tamponade is a life-threatening process in children admitted to the PICU especially after cardiac surgery. FCU allows for rapid evaluation of pericardial effusion (39). Evidence of effusion is typically most visible in a subxiphoid four chamber view. In the presence of effusion, tamponade can be sensitively indicated by right atrial collapse (42, 43). A study by Conlon et al., showed that credentialed pediatric intensivist-performed POCUS had a 95% concordance rate for the evaluation of pericardial effusion with a pediatric cardiologist (38). In another study by Spurney et al., the presence or absence of pericardial effusion diagnosed by pediatric intensivists with bedside echocardiogram was diagnosed with 91% accuracy (40). Ultrasound guidance in pericardiocentesis decreases complications and enhances first attempt success (48). Subxiphoid and apical approaches are preferred and the drainage is performed either in the short/out-of-plane axis or long/in-plane axis (49).

FCU in the PICU changes diagnosis and management (2, 50). Arnoldi et al. recently demonstrated that the incorporation of FCU in patients with presumed septic shock changed the intensivists' understanding of hemodynamics in 67% of the patients, suggesting that alignment of clinical management with a cardiac hemodynamic algorithm may improve outcomes in children with suspected septic shock (51). Additionally, a recent pilot study in adults demonstrated that critical care ultrasound-guided, goal-directed therapy in the setting of septic shock resulted in improved clearance of lactic acid at 6 hours and decreased fluid infusion volume at 12 and 24 h compared to the standard, early goal-directed therapy (52).

Finally, the purpose of hemodynamic POCUS is not to replace the formal echocardiogram (5). Rather, it uniquely equips the trained intensivist to answer focused questions with images and interpretations in the clinical context of the patient in the moment (43). It is best used as an adjunct to the physical exam and in conjunction with other means of assessing hemodynamic function that are available to intensivists (43). With the expansion of hemodynamic POCUS in pediatric critical care, there are now numerous guidelines and algorithms available to guide the focused application of echocardiography (3, 39, 53, 54).

### Lung Ultrasound

Acute respiratory failure, often secondary to pneumonia, bronchiolitis, and asthma exacerbation is the most common reason for admission to the PICU with pneumonia being the leading cause of death in children worldwide (55). The most commonly used radiographic test, standardized by the World Health Organization is the chest X-ray (CXR) (56). However, the CXR has been found to have relatively low sensitivity and specificity in differentiating etiologies in pediatric acute respiratory failure, suggesting the need for a tool with better diagnostic values. Lung ultrasound is a rapid, radiation free modality and when performed with a focused assessment, allows the clinician to rule in or out quickly and accurately diagnose certain clinical conditions (57).

Due to the high degree of impedance between soft tissue and air, well-aerated lungs are not well-visualized on ultrasound. Many pulmonary disease processes develop adjacent to the pleura and involve increasing pulmonary fluid or consolidations which provide enhanced ultrasound transmission, and cause alteration or disappearance of normal artifacts in pathologic ultrasound patterns (4). As a result, much of lung ultrasound relies on the interpretation of artifacts. These principles provide the foundation for lung ultrasound. To date, numerous protocols have been published, including the BLUE protocol which provides a standard approach for image acquisition and an algorithmic methodology to synthesize interpretation (58). Lung ultrasound can be used in the pediatric critical care to detect pneumonia, pneumothorax, pleural effusions, lung edema, and atelectasis, as well as guidance for chest tube placement and thoracentesis (3).

#### Evaluation of Lung Parenchyma

**Figure 4:**
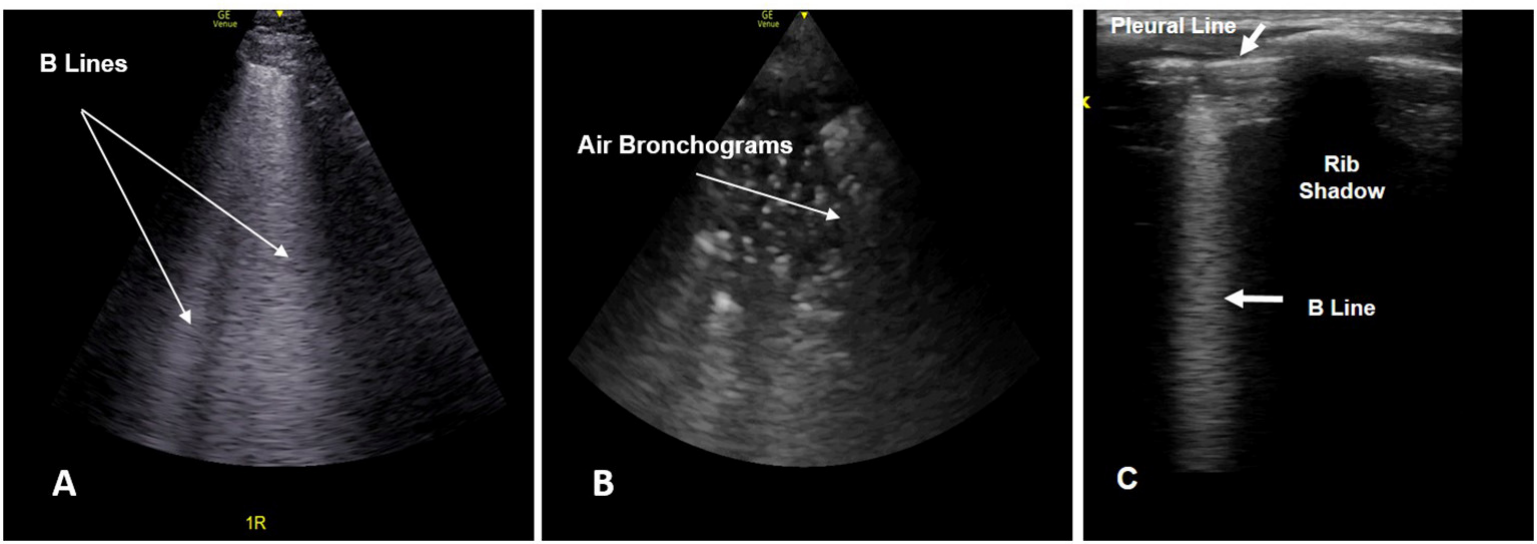
**(A)** Lung ultrasound image showing multiple B lines (arrow). **(B)** Lung ultrasound image showing air bronchograms (arrow) giving a “speckled” appearance. **(C)** Lung ultrasound image obtained using a linear probe showing a single B line.

In the evaluation of parenchymal lung disease, a variety of lung ultrasound patterns (pleural line abnormalities, consolidation, dynamic air bronchograms, and sometimes pleural effusion) aid in the diagnosis of pneumonia. B-lines (Figure 4) are vertical reverberation artifacts that indicate increased interstitial lung density. The distribution of B-lines have been shown to correspond with sub-pleural thickened interlobular septa, and are absent under normal conditions. More than two B-lines in a given ultrasound field is considered pathologic and indicative of alveolar-interstitial disease processes.

Lung ultrasound has a much higher diagnostic accuracy compared to CXR in the diagnosis of pneumonia with a pooled sensitivity of 96% and specificity of 93% in pediatric patients (57, 59). Recently, the severity of SARS-COV-2 pneumonia as determined by lung ultrasound performed by intensivists based on alveolar and interstitial consolidation was found to have a strong association with severity as assessed by chest CT (60). Lung ultrasound is also useful in the evaluation of other lung diseases such as bronchiolitis and atelectasis. Studies have demonstrated the ability for users to differentiate pneumonia from bronchiolitis and atelectasis with >85% sensitivity and specificity (61, 62). Lung ultrasound can also predict the need for oxygen and prognosis in patients with bronchiolitis presenting to an emergency room (63).

#### Evaluation of Pleural Space

**Figure 5:**
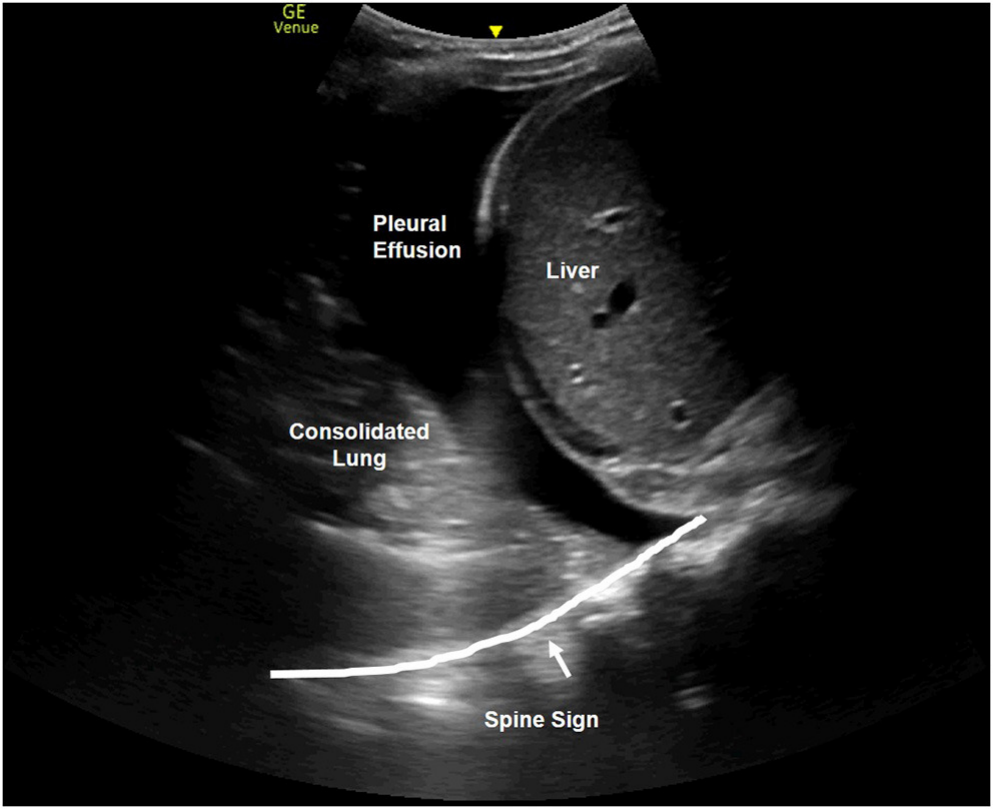
Right upper quadrant view of thoracic-abdominal cavity demonstrating liver, pleural effusion, consolidated lung and the spine sign.

Lung ultrasound detects pneumothorax in adults with a sensitivity and specificity >90%; (58) and in neonates with 100% sensitivity and specificity (64). The ultrasound pattern seen in pneumothorax involves an absence of lung sliding at the pleural line, which can be further elucidated in motion (M) – mode, the presence of A-lines, the absence of B-lines, and the visualization of a lung point. Lung point is a specific indicator of pneumothorax characterized by segments of lung sliding and abolished lung sliding in the same ultrasound image (58). Lung ultrasound is also the gold standard for the diagnosis of pleural effusion (Figure 5); it detects smaller volumes of pleural fluid compared to CXR and spares radiation associated with CT (65). Ultrasound guided evacuation of pneumothorax and pleural effusion is recommended in neonates, children, and adults to improve success of the procedure and to limit complications (3, 66, 67). While real-time ultrasound guided pleural fluid drainage is safe and easy, the free air associated with pneumothorax makes direct needle and landmark visualization difficult due to poor ultrasound transmission. Therefore, ultrasound is best employed as static guidance in pneumothorax evacuation (67). The use of ultrasound guidance in identification of thoracic landmarks prior to performing thoracentesis or the use of ultrasound guidance for real time thoracentesis in adult patients have been shown to decrease complications such as pneumothorax, inadvertent placement into the abdominal viscera and failed attempts (68, 69). The procedure can be safely performed even in patients on mechanical ventilation with a low rate of pneumothorax (70).

### Diaphragm Ultrasound

The diaphragm is easily identified on ultrasound due to its curved, dome-like muscular structure lined superiorly and inferiorly by parietal pleura and peritoneum, respectively. Diaphragmatic ultrasound has been a recent area for innovation with important critical care applications. Much of the work done involves diaphragmatic thickness and diaphragm thickening fraction. Diaphragmatic thickness is a static measure of the distance between the pleural and peritoneal layers. Thickening fraction of the diaphragm, which is used to assess diaphragmatic contractility, is the increase in thickness during inspiration expressed as a percentage (71, 72).

Diaphragmatic dysfunction is a loss of the muscular force generation of the diaphragm that has been associated with longer duration of mechanical ventilation and extubation failure (71–73). Ultrasound determined diaphragmatic thickness and thickening fraction has shown that diaphragmatic atrophy is greater in patients on neuromuscular blockade and that diaphragmatic contractility was linearly correlated with patients' degree of spontaneous breathing (72, 74). Ultrasound determined diaphragmatic atrophy has also been proven to be associated with prolonged post-extubation non-invasive positive pressure ventilation (73). These studies demonstrate the utility of diaphragmatic ultrasound as a tool to help identify patients who are at risk for diaphragmatic dysfunction-mediated morbidity. Similarly, in two pediatric studies, the investigators found an association between diaphragmatic thickening fraction during spontaneous breathing trials and successful extubation (75, 76). Diaphragmatic ultrasound has also been used in children to predict outcomes in both pneumonia and bronchiolitis using various diaphragmatic ultrasonographic metrics (77, 78).

### Abdominal Ultrasound

**Figure 6:**
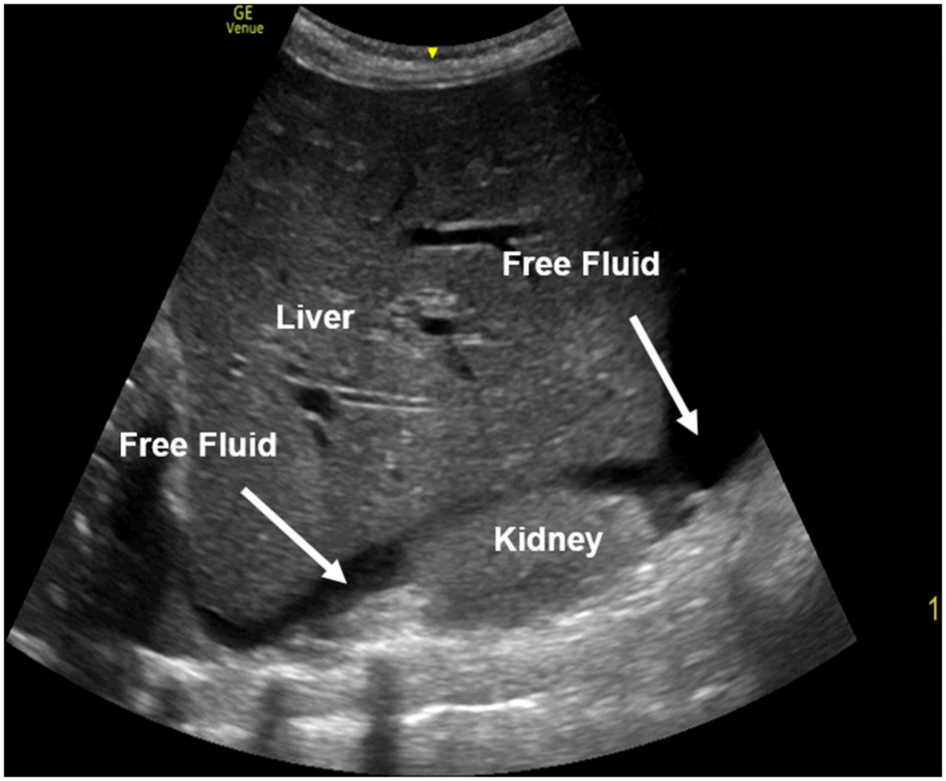
A right upper quadrant view performed in a FAST exam with free fluid present between the liver and the kidney. The free fluid appears black (anechoic) on ultrasound.

The focused assessment with sonography in trauma (FAST) examination is one of the original applications of resuscitation ultrasound, particularly in the emergency department (ED). It assesses for the presence of free fluid in the peritoneal cavity, and may be useful in the serial evaluation of blunt abdominal trauma in pediatric patients who are hemodynamically stable (79) (Figure 6). FAST exam has been shown to decrease the time to intervention in adult patients with blunt abdominal trauma (80). However, in a large randomized controlled trial of 925 pediatric patients treated in an ED following blunt torso trauma, the use of FAST compared with standard care only did not improve clinical care nor did it change the length of ED stay (81). In this study, there was no harm to the patients, including delay in care, missed findings or increased in number of computed tomography (CT) scans obtained because of the FAST exam. Since the FAST exam can be performed rapidly and repeated serially without much risk, it is prudent for providers to develop expertise in FAST even when the expected yield might be low. The traditional FAST has also been augmented to evaluate the lungs for hemothorax and pneumothorax, known as the extended FAST examination (eFAST) (82).

Point-of-care abdominal ultrasound can also be used for rapid estimation of bladder volume in oliguric/anuric critically ill children. Although it underestimates the true bladder volume compared to bladder catheterization (83), abdominal ultrasound is more accurate and reliable than the portable automated bladder ultrasound devices (84), especially at low bladder volumes. Use by bedside nurses is also a feasible approach, making this tool easily accessible for use (85).

Ultrasound guidance is frequently used in performing paracentesis and placement of abdominal drains. Mercardi et al., found that ultrasound guided paracentesis decreased the rate of complications such as bleeding and the associated patient care costs (68).

### Airway Ultrasound

Airway ultrasound is a novel point of care application in pediatric critical care (86). Clinicians are often challenged with questions such as vocal cord dysfunction, prediction of post-extubation stridor, anticipation of a difficult airway, and correct size and depth of endotracheal tube placement that are inadequately answered by current imaging or require invasive procedures.

The diagnosis of vocal cord dysfunction requires a flexible scope that is not always readily available and can be uncomfortable for the patient. A single view of the vocal cords obtained using bedside ultrasound can be used to diagnose vocal cord dysfunction in patients. A recent meta-analysis of eight observational studies, including 290 pediatric patients showed robust test characteristics of using bedside ultrasound to diagnose vocal cord dysfunction: pooled sensitivity of 91%, specificity of 97%, and a diagnostic odds ratio 333.56 (87). Adaptation by clinicians from diverse backgrounds, methodological and equipment similarities, rapid learning curve and a low risk of bias makes this an attractive bedside procedure (87).

**Figure 7:**
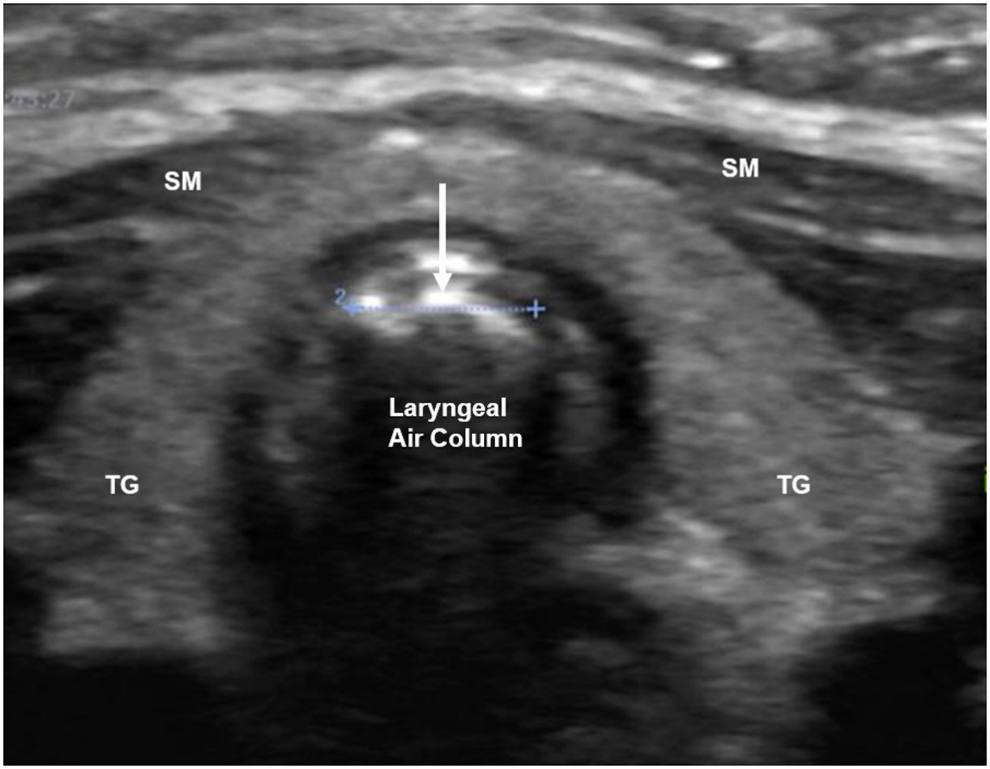
Airway ultrasound image at the level of thyroid gland demonstrating laryngeal air column width (arrow). TG, thyroid gland; SM, strap muscles.

Post extubation stridor complicates the clinical course of patients. Accurately predicting which patients will develop post extubation stridor allows clinicians to intervene both before and after development of stridor to minimize its clinical impact. Laryngeal air column width is measured using a single view obtained at the level of vocal cords (Figure 7). The difference in the width of the air column with the balloon cuff of the endotracheal tube inflated and deflated predicts post extubation stridor more accurately than the cuff leak test (accuracy 91% vs. 53%) (88).

Video laryngoscopy and the use of end tidal capnography are widely available and reliably predict endotracheal vs. esophageal intubation. However, when these are not available or in certain clinical scenarios, for instance in a patient with prolonged cardiac arrest or large pulmonary embolism when capnography is not reliable, bedside ultrasound can rapidly and accurately differentiate between esophageal vs. endotracheal intubation. A single view of trachea and esophagus at the level of cricothyroid membrane, while performing laryngoscopy and intubation predicts tracheal intubation with a sensitivity of 92–100% and a specificity of 100% (89, 90). The clinician can further measure the width of the air column at the level of cricoid cartilage to predict the correct size of cuffed (98% accuracy) and uncuffed (95% accuracy) endotracheal tubes (91).

Airway ultrasound can also be used to determine the depth of the endotracheal tube, and if the tube is in the right main stem bronchus. Because direct visualization of the endotracheal tube and the cuff is difficult (as they are both radiolucent), investigators have used indirect methods of cuff detection and depth of endotracheal tube placement. These include visualizing saline inflated cuff (sensitivity 98.8%, specificity 96.4%) (92), evaluating pleural sliding on both sides of the chest, (90) and assessing diaphragmatic movement on both sides simultaneously (93). Recently, clinical protocols combining these methods have been deployed with promising results in adults but validating studies are lacking in pediatrics (94). Although direct visualization of the endotracheal tube is feasible in neonates and infants, (95) this technique is much more challenging and only recommended for providers with experience in bedside ultrasound.

Airway ultrasound is promising in the prediction of a difficult laryngoscopy. A recent meta-analysis found robust test characteristics for prediction of a difficult laryngoscopy with significant sensitivity and specificity in adult patients using ultrasound (96). Ultrasound metrics evaluating anterior neck soft tissue thickness and mobility of the neck were found to be important predictors of a difficult laryngoscopy (96). To date, only one pediatric study evaluating ultrasound for difficult laryngoscopy has been performed that demonstrated good sensitivity (100%) and negative predictive value (100%) but with modest specificity (62%) and positive predictive value (19%) (97).

### Neurosonology

**Figure 8:**
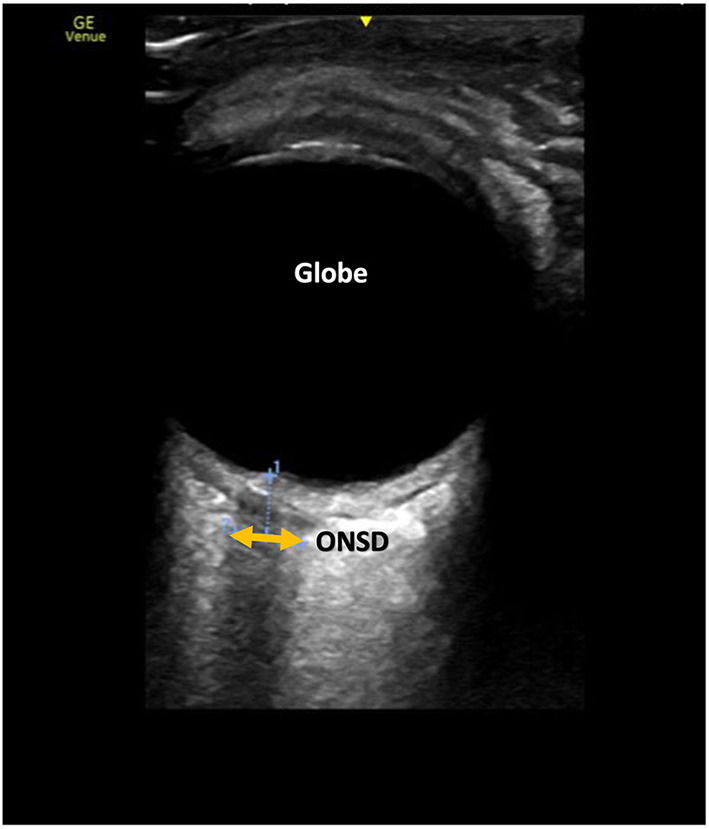
Ocular ultrasound demonstrating optic nerve sheath diameter measurement (ONSD). The sheath appears as a less bright (hypoechoic) structure compared to the surrounding tissue.

Traumatic brain injury is associated with significant morbidity and mortality in patients admitted to the PICU. Improving diagnosis and non-invasive monitoring of increased intracranial pressure and adequacy of cerebral perfusion remain attractive targets for clinical use and future research in neurocritical care (98). The two most common measurements used in cerebral ultrasound are the optic nerve sheath diameter (ONSD) and transcranial doppler estimation (99). Optic nerve sheath communicates with the subarachnoid space and increased intracranial pressure is transduced from the space to the sheath thereby causing sheath distention (Figure 8). Ultrasound measured ONSD above a certain threshold can predict increased intracranial pressure. Currently, most pediatric studies use ONSD >4.5 mm to predict increased intracranial pressure, which is a metric derived from adult studies (100). Isolated measurements at the time of patient presentation have been used to predict increased intracranial pressure due to a wide variety of etiologies such as hydrocephalus, shunt dysfunction, cerebral malaria, meningitis, intracranial mass and traumatic brain injury in pediatric patients (101). In these diverse patient populations, ONSD estimation performs well with a high sensitivity (pooled sensitivity 93%) and modest specificity (pooled specificity 74%) (101). However, a wide variation in the optimal threshold of ONSD and a modest specificity have prevented its applicability to clinical practice. There are also concerns that the plasticity of the sheath changes over time in patients with chronic elevation of intracranial pressure. Recently, serial measurements of ONSD performed in pediatric patients with traumatic brain injury and invasive intracranial monitoring failed to demonstrate co-relation between ONSD and increased intracranial pressure (102). Further work indexing ONSD to pediatric head size needs to be done before this metric can be integrated in clinical settings.

Transcranial doppler (TCD) has been used in the care of children for over three decades. TCD relies on the estimation of peak systolic, end diastolic and mean cerebral blood flow velocities from large cerebral arteries. These measurements are then used to derive physiological parameters to answer relevant clinical questions. Its utility in the outpatient management of patients with sickle cell disease is well-known (103). Its utility in neurocritical care is limited to evaluation of cerebral autoregulation, increased intracranial pressure, cerebral vasospasm, midline deviation and brain death (104). The availability and the utilization of this novel point of care tool remains restricted to a few specialized centers with resources and expertise in TCD. A recent survey of 29 pediatric neurocritical care centers found 20 centers utilizing TCD in their clinical practice to guide patient management. However, the adaptation was limited by the presence of equipment and trained physicians capable of performing and interpreting these studies, and the lack of standardized protocols across institutions (105). Consensus recommendations on standardizing these practices have since been proposed (106), but much work needs to be done before this can be integrated into current critical care practice.

## Practical Pocus Considerations

PICU patients are typically compromised, at least to some extent, and can be fragile and difficult to position. Additionally, many are connected to ventilators, extracorporeal circuits, or any number of different monitors at their bedside. Taken together, these factors can pose barriers to adequate POCUS access and image acquisition. Over the last few years, as POCUS technology has improved, clinicians now have access to smaller and more portable machines, mitigating some of these ergonomic issues (1). However, in certain circumstances, optimal positioning cannot be achieved, and the clinician must be flexible and trained in obtaining important information in less than ideal situations. The clinician must also be prudent in determining when the effort of POCUS is not in the patient's best interest, and therefore should be avoided or delayed.

POCUS equipment also has the potential to be a significant fomite (107). Infectious organisms such as staphylococcus aureus, pseudomonas aeruginosa, and vancomycin-resistant enterococci have been cultured from POCUS equipment, (108–112) and the ability of SARS-CoV-2 to survive on plastic emphasize the potential infectious threat (113). The importance of preventing POCUS from causing harm to already critically ill patients cannot be understated. To that end, numerous professional groups have published POCUS disinfection guidelines (114–117). Aseptic cleaning techniques have been shown to significantly reduce infectious organism burden on ultrasound equipment (118, 119). Emphasis on and adherence to these practices are important to prevent the benefit of POCUS from being outweighed by infectious consequences.

## Current State of Training and Competency Standards

Bedside ultrasound educators and program directors from several pediatric critical care fellowship programs conducted a detailed needs assessment that laid the foundation for programmatic development in the U.S. (15). They highlighted five core elements: training, credentialing, image storage, documentation, and quality assurance. Universally accepted standards for these core elements are lacking due to limited availability of high-quality evidence. Most large and small fellowship programs in the United States utilize diagnostic ultrasound and 79% of these programs provide formal fellow ultrasound training. However, only a small number of programs have quality assurance and credentialing processes in place. Image storage and appropriate documentation were also limited in the surveyed programs. The consensus among the survey participants was that having all five core elements in place would facilitate more effective implementation of bedside ultrasound.

Currently, a variety of training programs and curricula serve the educational needs of pediatric critical care physicians and trainees. National, regional, and institutional courses and longitudinal educational series are available at multiple centers. These courses can be expensive and limited in their availability. There is also a dearth of published curricular resources, thus making bedside ultrasound implementation difficult for programs without extensive infrastructure and personnel in place. The European Society of Pediatric and Neonatal Intensive Care (ESPNIC) published expert consensus guidelines on bedside ultrasound applications defining the scope of pediatric and neonatal critical care physicians; however several of these recommendations are expert opinions supported only by moderate quality evidence (3). Lack of infrastructure, personnel, training opportunities and well-defined scope of practice are barriers to education and widespread adaptation of bedside ultrasound.

Assessment of competency in bedside ultrasound is critical to its safe and effective application at the bedside. The current standards for competency are often defined by the number of examinations performed and are guided by limited evidence. The American college of Emergency Physicians recommends 25–50 high quality scans in each POCUS domain, while the Society of Critical Care Anesthesiologists recommends a higher number at 50 exams per domain (120, 121). However, it is important to recognize that the learning curve is different for each learner. Multi modal assessment tools that avoid the “one size fits all” approach such as direct observations, written examinations, structured clinical examinations and periodic quality assurance image review and feedback are necessary to effectively evaluate competency (122).

Lastly, credentialing allows a clinician who has demonstrated competency to integrate a new skill into their practice. It ensures the maintenance of a standard of care for both the patients and the physicians. The lack of published guidelines has contributed to difficulties in establishing credentialing pathways. Institutions have used a collaborative approach to bring together the major stakeholders and provide oversight as well as develop credentialing pathways (5). One such example is the implementation of ultrasound curriculum in a large academic pediatric critical care unit by Conlon et al., (14). Experiences from other institutions in the future will help further strengthen the development of standardized credentialing pathways.

## Future Directions for Point of Care Ultrasound

POCUS is beneficial in skilled hands, and emerging evidence suggest improved outcomes with its deployment. However, there are several challenges to its safe and efficacious implementation. First, there is a lack of adequate training, competency standards and evidence-based scope of practice. In 2020, the Joint Commission on Accreditation of Healthcare Organizations and the Emergency Care Research Institute cited POCUS without appropriate oversight as a major health technology hazard (123). This emphasizes the need for training and certification standards, oversight processes, and imaging and interpretation protocols to prevent adverse outcomes associated with POCUS implementation (124). As standardization practices are being developed, it is important that they are pediatric critical care-specific. Uniformity across individual programs at a professional society level would serve both as a safeguard and as a tool to optimize performance (125, 126). Also, there is a large variability in the level of expertise, the scanning protocols used, the heterogeneity of the patients and clinical conditions, and the lack of systematic approach that prevents consistent integration into clinical practice.

Ultrasonographic technological advances will help POCUS innovation and implementation (1). Smaller, more versatile handheld ultrasound devices with Doppler, M-mode, and other quantitative abilities are more readily available (127–129). Convenience without compromising capability will improve application and consistent informed decision making. Artificial intelligence is also being applied to ultrasound technology to flatten the learning curve and improve image acquisition (1). Deep-learning algorithms applied to POCUS have the potential to be transformative as it allows the ultrasound machine to guide image acquisition (130) and to detect certain pathologies in point-of-care images (131, 132). Although emerging work in ultrasound artificial intelligence could be the driving force behind POCUS training and application advancement, it could also pose challenges to the development and enforcement of competency and safety measures.

## Conclusion

In its current state, pediatric critical care POCUS lags behind other areas of critical care in implementation and expertise. The application of training and certification standards in parallel with emerging technology will improve competency and confidence among clinicians (8). As more skilled practitioners become available, it will be important that an appropriate scope of practice is defined and applied. Evidence based pediatric critical care-specific literature to support and validate practices will be crucial (4, 9, 13). The ultimate goal of improving and increasing POCUS use in the specialty is not to increase the ultrasound footprint in pediatric critical care; but rather to optimize the care that patients receive in the PICU. A glossary of important terms used can be found in Table 3.

**Table 3:** Glossary of terms.

Air Bronchograms	Air filled bronchi surrounded by alveoli filled with fluid, pus or other material. These appear as alternate areas of bright and dark structures on ultrasound
Aortic Flow Variability (AFV)	Change in the velocity of blood flow during respiratory cycle, measured over the aortic valve
B-lines	Vertical artifact on lung ultrasound signifying pleural or parenchymal pathology
B-mode	Brightness Mode - standard ultrasound that generates 2-dimensional gray scale images
Doppler	Measurement of velocity and direction of moving structures using ultrasound
End Point Septal Separation (EPSS)	Assessment of mitral valve leaflet movement toward interventricular septum using Motion (M) - mode
Fractional Area Change (FAC)	The change in left ventricle area between systole and diastole expressed as a percentage
Fractional Shortening (FS)	The change in left ventricle diameter size between systole and diastole expressed as a percentage
Impedance (acoustic)	The resistance to the propagation of ultrasound waves through the tissue.
IVC Collapsibility Index (IVCCI)	The change in the diameter of IVC in a spontaneously breathing patient over the respiratory cycle
IVC Distensibility Index (IVCDI)	The change in the diameter of IVC in a mechanically ventilated patient over the respiratory cycle
Laryngeal Air Column Width (LACW)	The width of the column of air as determined by ultrasound
Longitudinal axis	The evaluation of a structure along its length. Also referred to as In-plane or long axis approach
Lung sliding	Dynamic movement seen on ultrasound at the pleural line as visceral pleura slides along the parietal pleura
M-mode	Motion mode - narrows to a single line of B-mode that permits a still image to demonstrate motion and allows for measurements of rapidly moving structures
Pulsed-wave doppler	Doppler principle of sending pulses of ultrasound and analyzing reflected sound waves between the pulses
Transverse axis	The evaluation of a structure in a plane orthogonal to its length. Also referred to as the out of plane or short axis approach.
Velocity Time Integral (VTi)	Doppler ultrasound measurement of blood flow. It is measured as the area under the velocity time curve obtained from doppler waveform

## Author Contributions

LB, VB, and MK contributed to the conception and writing and final edits of this manuscript. All authors contributed to the article and approved the submitted version.

## Conflict of Interest

The authors declare that the research was conducted in the absence of any commercial or financial relationships that could be construed as a potential conflict of interest.

## Publisher's Note

All claims expressed in this article are solely those of the authors and do not necessarily represent those of their affiliated organizations, or those of the publisher, the editors and the reviewers. Any product that may be evaluated in this article, or claim that may be made by its manufacturer, is not guaranteed or endorsed by the publisher.
